# Occurrence of multiple infections of rodents with parasites and bacteria in the Sibang Arboretum, Libreville, Gabon

**DOI:** 10.14202/vetworld.2024.2506-2516

**Published:** 2024-11-13

**Authors:** Patrice Makouloutou-Nzassi, Chimène Nze-Nkogue, Boris Kevin Makanga, Neil Michel Longo-Pendy, Judi Armel Bourobou Bourobou, Branly Cordia Bikie Bi Nso, Etienne François Akomo-Okoue, Cherone-Cheba Mbazoghe-Engo, Félicien Bangueboussa, Silas Lendzele Sevidzem, Ghislain Wilfried Ebang Ella, Lillian B. Mangama Koumba, Fred Loïc Mindonga Nguelet, Rodrigue Mintsa Nguema, Larson Boundenga

**Affiliations:** 1 Department of Biology and Animal Ecology, Institut de Recherche en Ecologie Tropicale (IRET/CENAREST), BP 13354 Libreville, Gabon; 2 Unité de Recherche en Ecologie de la Santé, (URES/CIRMF), BP 769, Franceville, Gabon; 3 Department of General Agronomy, Institut de Recherches Agronomique et Forestière (IRAF/CENAREST) BP 2246, Libreville, Gabon; 4 Department of Health and Environment, Université Libreville Nord, BP 1177 Libreville, Gabon; 5 Department of Anthropology, Durham University, South Road, Durham DH1 3LE, UK

**Keywords:** *Anaplasma*, Babesia, Gabon, *Haemaphysalis*, Helminths, *Laelaps*, *Praomys*

## Abstract

**Background and Aim:**

Rodents are carriers or reservoirs of various bacteria, protozoa, viruses, and ectoparasites. Given the proximity of various rodent species and humans, there is a potential for the transmission of pathogens. Data on ecto- and endo-parasite prevalence in rodent populations in Gabon are limited. To fill this gap, we conducted a study in Libreville to investigate the occurrence of ecto- and endo-parasites in rodents.

**Materials and Methods:**

We captured and euthanized 68 rodents belonging to the genus *Praomys* and examined their ecto- and endo-parasite fauna, dissected their gastrointestinal tract for helminths, and prepared blood smears to examine blood-borne pathogens.

**Results:**

Our analyses identified three pathogen taxa: helminths (*Protospirura* spp., *Trichuris* spp., and *Taenia* spp.), protozoa (*Babesia* spp.), bacteria (*Anaplasma* spp.), and arthropods (*Laelaps* and *Haemaphysalis*). Overall, 91.2% of the rodents were infected with at least one pathogen and ectoparasite, with helminth occurrence rate of 63.2% and ectoparasite occurrence at 44.1%. Protozoan infections (*Babesia* spp.) were found in 10.3% of the rodents, whereas bacteria (*Anaplasma* spp.) had an occurrence rate of 39.7%.

**Conclusion:**

Native rodents in Libreville harbor various infectious agents, ecto- andendo-parasites. These findings highlight the potential health risks associated with *Praomys* rodents for the transmission of various diseases to human population in Gabon and emphasize the need for investigation of rodents for their role as disease carriers.

## Introduction

Rodentia comprises the largest and most diverse group of living mammals, accounting for approximately 43% of all mammalian species [1]. They play key ecological roles through their burrowing activities [2]. Rodents are globally recognized as competitors with humans for food, primarily due to the preharvest damage they cause to cereals [3], resulting in billions of dollars in property and food resource losses annually [4]. Rodents are second to bats in carrying zoonotic pathogens [5–7]. Throughout history, rodents have been implicated in the spread of numerous diseases, such as plague [8, 9], murine typhus [10, 11], and leishmaniasis [12, 13]. Recent studies have shown that more than 10% of global rodent populations carry or serve as reservoirs for pathogens, which have public health implications [14, 15].

In Gabon, rodents were found to carry bacteria (*Anaplasma* spp., *Bartonella* spp., *Coxiella burnetii*, and *Leptospira*) [16, 17], protozoa (*Plasmodium*, *Theileria* spp., *Toxoplasma gondii*, and *Trypanosoma*) [16, 18, 19], and viruses (Astroviruses, Orthonairoviruses, and lymphocytic choriomeningitis virus) [20–23]. These stu-dies were conducted in six of the nine provinces of Gabon. However, there is limited knowledge regarding the helminths and ectoparasites that are circulating among rodent populations. A study in the Moukalaba-Doudou National Park focused on helminth parasites in rodents [24].

Our study aimed to investigate the occurrence of pathogens and ectoparasites that can be hosted by rodents in Libreville. In addition, we aimed to assess the impact of host sex and age on the prevalence and diversity of rodent pathogens in urban settings.

## Materials and Methods

### Ethical approval

The rodents were captured with theagreement of the mayor of the district through Letter no. 08/M6/LBV and the head of the Institut de Pharmacopée et Médecine Traditionnelle (IPHAMETRA/CENAREST) in charge of the study site. All animal specimen collection procedures complied with applicable institutional and national guidelines for the use and handling of animals.

### Study period and location

**Figure-1:**
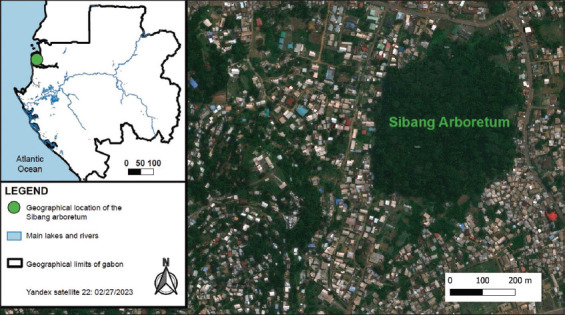
Map of the study area showing the Sibang arboretum where rodent capture occurred [Source: The map was generated using QGIS 3.38.3 with Yandex Maps satellite].

This study was conducted in the SIBANG arboretum at 0°25’N and 9°29’E (Figure-1) during June and July 2020. Covering 16 ha, this forest park is in the eastern section of Libreville, the capital city, and is traversed by the Adoung River. Cordier [25] reported that the arboretum hosts 137 plant species and 40 bird species, although the diversity of vertebrates, including reptiles and small mammals, remains under-researched. Despite being a protected area, the Sibang Arboretum has been significantly affected by significant human impacts, including regular human activity and waste accumulation [26], illustrating the interactions between natural and altered environments.

### Trapping of rodents

In July 2020, rodents of different ages and sexes were captured using Sherman (H. B. Sherman Inc., Tallahassee, Flor.) and Tomahawk (U.S. Hwy 51, Hazelhurst, WI 54531, United States) traps baited with palm nuts, following line transects [27, 28]. A total of 138 traps were strategically set in six 220-m transects, with 23 traps placed on each side at 10-m intervals. Daily checks over a 3-day period resulted in a total trapping effort of four trap nights.

### Identification of rodents

For accurate identification, we used a standardized identification key based on the external characteristics of the captured rodents [29]. After capture, each rodent was transported to the Iphametra Laboratory. Following a pre-established protocol [30], the rodents were humanely euthanized using a halothane solution and then necropsied. To induce sleep, we placed the rodents in a plastic bag containing halothane-soaked cotton. Once asleep, they were carefully positioned on a white cloth for examination. We measured head-body, tail, ear, and hindfoot length. Species identification was based on keys by Wilson and Reeder [31], which included various measurements and pelage characteristics. We recorded the sex of each rodent, while age classification (adults or juveniles) was determined based on morphological features [32]. Ultimately, we identified all trapped rodents at the genus level, confirming that they belonged to the *Proamys* genus.

### Pathogen screening

The skin and gastrointestinal (GI) tract were carefully examined for ecto- and endo-parasite using a stereomicroscope. Ectoparasites were collected following the method of Kia *et al*. [33] for each rodent and preserved in 0.5 mL of 95% ethanol in an Eppendorf tube (Eppendorf AG, 22331 Hamburg, Germany) at room temperature (25°C) for identification. The collected ectoparasites were morphologically identified at the genus level using methods previously developed by Tomlinson *et al*. [34], Dwużnik *et al*. [35], and Savchenko and Lareschi [36]. Blood was collected from the heart post-dissection using needle syringes, thin blood smears were prepared, stained with Giemsa solution, and allowed to dry for parasite detection and identification. The GI tract was dissected and examined for parasites after draining contents into physiological saline using the method described by Paperna [37]. GI helminths were identified at the genus level using keys [38, 39] under a microscope (Leica DM, Danaher, USA) at 400× magnification. Hemoparasites and bacteria were detected and identified at 1000× magnification following relevant keys [40, 41].

### Statistical analysis

Descriptive statistics (Table-1) were first computed to summarize the captured rodents and provide the parasites found on them. Next, quantitative descriptors of the parasites, including occurrence rate (OR), the ecological index of dominance (D), and the mean abundance (MA), were calculated (Tables-2 and 3) as described by Bush *et al*. [42]. The OR indicates the proportion of infested hosts, calculated using OR = (n/Z)*100.

**Table-1:** Characteristics of the rodent population.

Sex	Age status	Number	Min. weight (g)	Max. weight (g)	Mean weight (SD)
Female	A	26	18	59	31.77 (7.44)
	J	2	12	18	15 (4.24)
	A & J	28	12	59	30.57 (8.44)
Male	A	36	13	55	33.22 (7.66)
	J	4	13	30	20.5 (7.33)
	A & J	40	13	55	31.95 (8.47)
Total (Male and female)		68	12	59	31.38 (8.42)

SD=Standard deviation, A= Adult, J=Juvenile

**Table-2:** Number of infected rodents per pathogen taxa.

Sex	Number of infected rodents per pathogen							Sub-total

	Bacteria	BP	Ectoparasite		GI parasite			
Female	Ana	Bab	Hae	Lae	Tae	Pro	Tri	
A	7	1	3	9	11	3	10	22
J	2	1	0	0	1	0	0	2
A & J (%)	9 (33.3)	2 (28.6)	3 (4.4)	9 (13.2)	12	3	10 (45.5)	24 (35.3)
Male								
A	15	5	12	16	15	5	11	34
J	3	0	1	1	2	1	1	4
A & J (%)	18 (66.7)	5 (71.4)	13 (19.1)	17 (25)	17	6	12 (54.5)	38 (55.9)
Sub-total (%)	27 (39.7)	7 (10.3)	16 (23.5)	26 (38.2)	29	9	22	62 (91.2)

BP=Blood parasite, GI=Gastrointestinal, Ana=*Anaplasma* spp., Bab=*Babesia* spp., Hae=*Haemaphysalis* spp., Lae=*Laelaps* spp., Tae=*Taenia* spp., Pro=*Protospirura* spp., Tri=*Trichuris* spp., A= Adult, J=Juvenile

**Table-3:** Infestation parameters of pathogens in *Praomys* rodents collected from the Sibang Arboretum, Gabon.

Quantitative descriptors	Ectoparasite		GI parasite			Bacteria	BP
			
	Hae	Lae	Tae	Pro	Tri	Ana	Bab
NPR	16	26	29	9	22	27	7
NAR	52	42	39	59	46	41	61
NP (M, F)	106 (0, 106)	74 (1, 73)					
OR (%)	23.5	38.2	42.7	13.2	32.4	39.7	10.3
D (%)	58.9	41.1					
MA	1.56	1.09					
OR (%)	45.6		63.2			39.7	10.3

NPR=Number of presences on rodents, NAR=Number of absences on rodents, NP=Number of parasites, M=Male, F=Female, OR=Occurrence rate, D=Ecological index, MA=Mean abundance, BP=Blood parasite, GI=Gastrointestinal, Ana=*Anaplasma* spp., Bab=*Babesia* sp., Hae=*Haemaphysalis* spp., Lae=*Laelaps* spp., Tae=*Taenia* spp., Pro=*Protospirura* spp., Tri=*Trichuris* spp.

Where n is the number of infested *Praomys* rodents and Z is the total number examined. The descriptors D and MA were calculated for ectoparasites because two species were identified and parasite intensity was assessed. Dominance (D) was computed using D = (Ec/Ep) 100.

Where, Ec is the number of ectoparasites in a given species and Ep is the total number of ectoparasite species collected. D represents the relative abundance of a species within a community.

MA was calculated using MA = Ea/Ht,

Where Ea is the number of ectoparasites in a specific species and Ht is the total number of hosts examined.

All quantitative descriptors and descriptive statistics were computed using Excel 2016 (Microsoft Corporation, Washington, USA). Fisher’s exact test was used to analyze the significance of differences in prevalence [43]. Finally, the correlation between infection status by type of infection status (General infection, multiple infection, gastro-intestinal parasitism, blood parasitism, ectoparasitism, and bacterial infection) and independent variables (Rodents age, rodents’ sex, and rodents weight) was computed using specific tests in R software R.4.3.2 (R Foundation for Statistical Computing, Vienna, Austria). For example, Fisher’s exact test was used to assess the association between two categorical variables (e.g., multiple infection status vs. rodent sex), while the two-sample Wilcoxon rank sum test was used to evaluate correlations between dichotomous categorical variables and continuous variables (e.g., General infection vs. rodent weight). The Kruskal–Wallis test was applied to analyze correlations between non-dichotomous categorical variables and a continuous variable (e.g., Multiple infections vs. rodent weight).

## Results

### Characteristics of the captured rodents

Sixty-eight rodents from the *Muridae* family were captured during the study; all were identified as *Praomys* sp. Of these, 28 were female (41.18%) and 40 were male (58.82%). The majority of rodents were adults, totaling 62 individuals (91.2%), while only six were young (8.8%) (Table-1).

### Parasite infection parameters of *Praomys* rodents collected from the Sibang Arboretum, Gabon

Three pathogen taxa were identified in the captured rodents: helminths (*Protospirura* spp., *Trichuris* spp., and *Taenia* sp.), protozoa (*Babesia* spp.), bacteria (*Anaplasma* spp.), and arthropods (*Laelaps* and *Haemaphysalis*). No fleas or lice were present. Approximately, 62 (91.2%) of the captured rodents were infected with at least one pathogen or ecto/endoparasite, with no significant difference in occurrence between infected females (24, 85.7%) and males (38, 95%) (p > 0.05).

#### Ectoparasite occurrence

Out of the 68 captured rodents, 31 (45.6%) were infested with at least one ectoparasite, yielding 180 individual ectoparasites (mean 2.65 ± 7.40 per infested rodent) (Table-3). Ticks were the predominant ectoparasite, constituting 58.9% of the ecological index but only 23.5% of infestations. In contrast, mites were found in 38.2% of the infestations, despite being less abundant.

**Figure-2:**
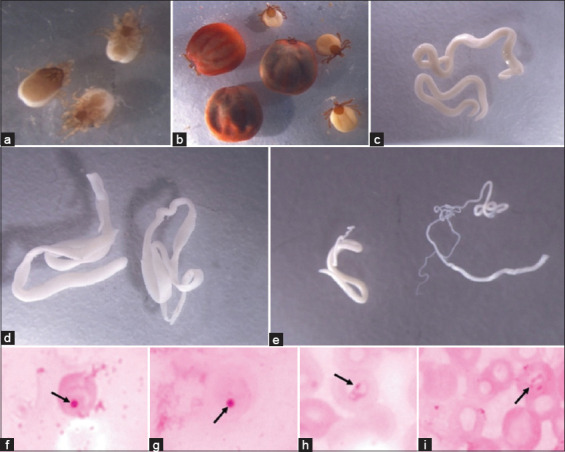
Micrographs of infectious agents recovered from Gabonese rodents in the Sibang Arboretum: (a) *Laelaps* spp., (b) *Haemaphysalis* spp., (c) *Protospirura* spp., (d) *Taenia* spp., (e) *Trichuris* spp., (f and g) *Anaplasma* spp., and (h and i) *Babesia* spp.

All collected ticks (n = 106) were identified as *Haemaphysalis* spp. (Figure-2b), and only females were present (Table-3). Male rodents had higher infestation rates (19.1%) than females (4.4%) (p %0.05) (Table-2).

Among the mites, only one Mesostigmatic mite, *Laelaps* spp., was identified (Figure-2a), with infestation rates of 25% in male rodents and 13.2% in females (p > 0.05) (Table-2). In total, we collected 74 mites, including 73 females (98.6%) and one male (1.4%). The MA of *Haemaphysalis* spp. (1.56) slightly exceeded that of *Laelaps* spp. (1.01) (Table-3).

#### GI parasites, bacteria, and hemoparasites

Of the 68 rodents examined, 43 were infected with helminths (GI parasites), resulting in an occurrence rate of 63.2% (Table-3). The identified helminths included two nematode species, *Protospirura* (Figure-2c) and *Trichuris* (Figure-2e), along with the cestode *Taenia* (Figure-2d). *Taenia* was responsible for 42.6% (29/68) of infections, whereas *Trichuris* had an occurrence rate of 32.3% (22/68) (Table-3). Adult *Trichuris* parasites were characterized by thin anterior and thicker posterior ends. The occurrence of *Trichuris* infection was slightly higher in males (54.5%) than in females (45.5%), but the difference was not statistically significant (Table-2) (p > 0.05). One juvenile out of 21 adults was also infected with *Trichuris* (Table-2). In addition, the protozoan parasite *Babesia* (Figures-2h and i) and the bacterium *Anaplasma* (Figures-2f and g) were isolated from the rodents. In total, 7 rodents (10.3%) were infected with *Babesia* spp., consisting of 5 males (71.4%) and 2 females (28.6%). Twenty-seven rodents were found to be infected with *Anaplasma* spp. (39.7%), including 18 males (66.7%) and 9 females (33.3%) (Table-2).

#### Multiple infections

In total, 28 (45.2%) of the rodents in this study had multiple pathogens and ectoparasites. Among the 62 rodents that harbored pathogens, ecto- and endo-parasites, 17 (26.5%) had dual infections, 7 (10.3%) had three concurrent infections, and 4 (5.9%) had four simultaneous infections (Table-4). Table-4 presents an overview of the identified coinfection.

**Table-4:** Diversity of co-infection combinations of pathogens in *Praomys* rodents collected from the Sibang Arboretum in Gabon.

Co-infection	OR (%)	Times	Species richness
Arthropods + Bacteria	26.5	1	Hae + A
Arthropods + Helminths	26.5	7	L + T + Tr; L + Tr; Hae + L + Pr; L + Tr; Hae + L + Pr; Hae + L + Tr; L + Tr
Bacteria + Helminths	26.5	7	A + T + Pr; A + T; A + T + Tr; A + T; A + T; A + Pr; A + Tr
Bacteria + Protozoa	26.5	2	A + B; A + B
Arthropods + Bacteria + Helminths	10.3	7	Hae + L + A + Pr; Hae + L + A + T + Tr; L + A + T + Pr; L + A + T; Hae + L + A + T + Tr; L + A + Tr; Hae + L + A + Tr
Arthropods + Bacteria + Helminths + Protozoa	5.9	4	L + A + Tr + B; Hae + L + A + T + Tr + B; Hae + A + T + Tr + B; Hae + L + A + Tr + B
OR (%)	45.2		

A=*Anaplasma*, B=*Babesia*, L=*Laelaps*, Hae=*Haemaphysalis*, T=*Taenia*, Tr=*Trichuris*, Pr=*Protospirura*, OR=*Occurrence* rate

### Effects of age, sex, and weight on the infection status of the rodents

There were generally no significant correlations between infection status and rodent characteristics (p > 0.05; Table-5), indicating independence. However, a significant relationship exists between bacterial infection status and rodent age (p = 0.03294; Table-5).

**Table-5:** Correlation analysis between infection status and the characteristics of the rodents (independent variables).

Infection type	Rodent sex	Rodent age	Rodent weight
		
	p-value	p-value	p-value
General infection	0.2203	1	0.1408
Multiple infection	0.4841	0.4699	0.1042
Gastro-intestinal parasitism	1	1	0.2788
Blood parasitism	0.6912	0.4927	0.8319
Bacteria infection	0.3234	**0.03294**	0.1271
Ectoprasitism	0.4616	0.2089	0.1808

In bold, p < 0.05 indicates statistical significance

## Discussion

This study provides a comprehensive analysis of ecto- and endo-parasites, as well as bacteria, found in a native rodent species (*Praomys* sp.) captured in the Sibang arboretum. This *Muridae* family rodent is typically found in Guinean Congolese forests, including the Sibang arboretum in Libreville, Gabon. The African *Muridae* rodents are predominantly found in savannas and forests to the south of the Sahara subdesert zone. The *Praomys* genus thrives in forested and degraded areas near human habitats, such as the Sibang arboretum [44, 45]. The study observed that these indigenous rodent species frequently exhibited multiple concurrent infections with arthropods, bacteria, helminths, and protozoa, confirming that wildlife often experiences such coinfections [46–48]. Therefore, the correlation between host-related parameters (age, sex, and weight) and infection status was evaluated. Multiple coinfections can affect both the physical condition and immunity of hosts [49–51]. However, in this study, none of the factors were analyzed as potential determinants of infection status in Libreville rodents, except for animal age. We found that adult rodents had the highest infection rates (Table-5), consistent with previous studies by Taylor *et al*. [52], Vanden Broecke *et al*. [53], and Mariën *et al*. [54], probably due to their roaming behavior and the pre-patent period [55, 56]. In addition, males exhibited a higher infection rate than females, a pattern observed in other studies of rodents across Africa and beyond [57–59]. This difference indicates that GI parasitism in these animals probably depends on sex and can be attributed to factors such as grooming behaviors, roving tendencies [60–62], large home ranges [63, 64], and higher testosterone levels, which are linked to increased stress and reduced immunity [65, 58].

In our study, we identified two ectoparasite arthropods: ticks and mites. The ectoparasite species diversity was low, likely due to the small number of rodent hosts examined, with fewer than 100 rodents screened for ectoparasites. Contrarily, previous studies by Mawanda *et al*. [45] and Babyesiza *et al*. [66] have reported multiple ectoparasites in *Praomys*. These variations are linked to host ecological traits, immune responses, and specificity [67–70].

Although the less prevalent, *Haemaphysalis* species was abundant in our study, and it is documented in Cote d’Ivoire [71], Mali [72], and Senegal [73]. We did not identify the species. However, a study conducted in Gabon revealed *Haemaphysalis leachi* ticks, but not in rodents [74]. The *Laelaps* ectoparasite identified in our study has not been previously recorded in Gabon, although it has been documented in other African countries where species identification has occurred [45, 75–77]. *Laelaps* species are highly prevalent, and previous studies reported by Mawanda *et al*. [45] and Matthee *et al*. [76] have indicated their presence across various hosts. Some Laelapid mites are generalists that can parasitize various rodents, facilitating their survival and spread [78].

Our study found a high occurrence rate of GI parasites (63.2%) in examined rodent samples, consistent with findings by Mafiana *et al*. [79] in Nigeria (64.7% in *Rattus rattus*) and Elshazly *et al*. [80] in Egypt (52.8% in domestic rodents). However, our occurrence rate is higher than the 27% reported by Asakawa and Nicolas [24] in Gabonese wild murids, the 15.5% in spiny mice (*Acomys spinosissimus*) by Lutermann *et al*. [81] in South Africa, and the 14.7% in Ansell’s mole-rat, (*Fukomys anselli*) by Lutermann *et al*. [82]. These variations may stem from ecological factors and anthropogenic disturbances. The study observed a high prevalence in *Praomys* sp., a rodent species broadly distributed in its habitat that is vulnerable to parasites [83]. Only three helminth taxa (*Protospirura*, *Trichuris*, and *Taenia*) were identified, indicating low diversity compared to seven species found in Nigeria [79], and 24 found in Egypt [80], underscoring ecological variation. Our research focused on forest-trapped rodents, unlike previous studies [79, 80] that involved synanthropic rodents. However, the low parasite diversity in wild Gabonese rodents is not clear. Asakawa and Nicolas [24] identified only one nematode species, whereas our study isolated three helminth species. Like Asakawa and Nicolas [24], we identified the spirurid nematode species, *Protospirura* spp., in our murid rodent species. Although we were unable to identify the species based on morphological features alone, the species was isolated from the same host rodent species: *Praomys* sp.

In addition, Asakawa and Nicolas [24] did not identify whipworms; thus, our study is the first to isolate this parasite from wild Gabonese murids. However, we could not determine the species based on morphological criteria alone. The previous studies by Mafiana *et al*. [79] and Elshazly *et al*. [80] morphologically identified the rodent whipworm species *Trichuris muris*. Ribas *et al*. [84] employed molecular tools to identify *T. muris*. Instead, the authors discovered two novel whipworm species in West African rodents, alongside *Trichuris mastomysi*. *Trichuris* (whipworms) are nematodes that are primarily found in the GI tract. Although various species of *Trichuris* infect humans, we consider them to be largely classic due to their abundance in humans and pigs [85–87]. Other species, especially those in rodents, could potentially become zoonotic if they adapt to humans. The presence of *Trichuris* in the rodents of Libreville suggests that they may act as reservoirs for whipworms, potentially contributing to the transmission of trichuriasis, particularly in cases of poor sanitation or close human-rodent interactions.

The present study may be the first to document an adult cestode tentatively identified as *Taenia* spp. in Gabonese wild murids; previous research reported by Asakawa and Nicolas [24] did not isolate this parasite. Although *Taenia* has been noted in African rodents, it was mostly documented in its larval form [79, 80, 88]. Martino *et al*. [89] found four adult cestode parasites in wild nutria (*Myocastor coypus*, Molina, 1782), including an unidentified *Taenia* spp. [90]. Given the difficulty of distinguishing taeniid species morphologically [91, 92], we plan to employ molecular tools to identify cestode parasites. *Taenia* comprises zoonotic and anthropophilic cestodes. Among the known species, *Taenia solium* is commonly found in swine [93], while *Taenia saginata* is associated with cattle [94]. Other species may also exhibit zoonotic transmission cycles. For instance, *Taenia taeniaeformis* is primarily hosted by felines [95], and it exhibits a transmission cycle involving rodents [96]. The presence of *Taenia* in the rodent population of Libreville warrants further investigation into the infecting species and their potential zoonotic impact, especially in an urban setting with frequent human-rodent interactions.

*Babesia* spp. has been detected in bovines in Gabon [99] but has not yet been reported in rodents. However, two studies in Africa reported its presence in rodents [97, 98], with prevalence rates of 1.7% and 38.7%, respectively. The first study employed morphological screening [98], while the second combined morphological and molecular analyses identifying a new *Babesia* species: *Babesia behnkei* sp. nov.[97]. Although the species identified in the present study remain unnamed, their detection suggests active transmission of *Babesia* by ticks in Gabon, as the parasite has been isolated in other animals [99]. However, there has been no investigation into its circulation among the human population [100]. *Babesia* species, especially *Babesia microti* and *Babesia divergens*, cause tick-borne disease called babesiosis in humans [101, 102]. Rodents act as reservoir hosts for these parasites, which can be transmitted to humans through tick bites [103, 104]. Their presence in the rodent populations of Libreville indicates the potential for zoonotic transmission.

Mangombi *et al*. [16] used molecular tools to demonstrate the presence of *Anaplasma* spp. in murid rodents in Franceville, Gabon, with a prevalence of 8.1%. In the present study, *Anaplasma* spp. was identified through morphological screening and exhibited a high infection rate of 39.7%. These variations could be attributed to ecological factors, although the species was not identified, and different rodent hosts were involved. This underscores the capability of pathogens like *Anaplasma* spp. to infect various hosts [105]. *Anaplasma phagocytophilum* is a known zoonotic pathogen that causes human granulocytic anaplasmosis [106, 107]. Rodents are recognized as primary reservoir hosts, and transmission to humans occurs primarily through infected tick bites [77, 108]. The detection of *Anaplasma* in rodents in Libreville indicates potential local transmission cycles that pose a zoonotic risk to humans. Public education on tick bite prevention and effective tick control methods are essential to mitigate this risk.

Although this study establishes a valuable baseline for future research on rodent pathogens in Gabon, it has limitations, including a lack of data on infection intensity (parasite load), except for ectoparasites, and its cross-sectional design restricts definitive conclusions.

## Conclusion

This study in Libreville investigated infectious agents in rodents, making it among the first such efforts in the country. Findings showed that native Gabonese rodents, specifically *Praomys* sp., harbor five infectious agents, including pathogenic agents such as *Taenia* spp., *Babesia* spp., and *Anaplasma* spp., alongside ectoparasites. These results indicate that various infectious agents in rodent populations pose a risk to humans, highlighting the need for systematic detection in human populations.

Although this study provides valuable insights, certain limitations need to be addressed, including low sampling power, short duration, and failure to identify rodent species and their pathogens. To address these gaps, follow-up studies will be conducted throughout all seasons with larger sampling efforts. Future research will also use molecular methods to characterize rodent species, their ectoparasites, and the pathogens that they may transmit to humans.

## Data Availability

The datasets used and/or analyzed during the current research are provided in the manuscript. Further details can be obtained from the corresponding author on reasonable request.

## Authors’ Contributions

PMN and CNN: Conceived and designed the study. PMN, CNN, BKM, BCBBN, EFAO, GWEE, LBMK, FLMN, GWEE, FB, and CCME: Conducted the study. PMN, NMLP, JABB, SLS, and LB: Analyzed the data. PMN, CNN, JABB, SLS, and GWEE: Drafted and revised the manuscript. LB and RMN: Supervised the study. All the authors have read and approved the final manuscript.
